# Improving care for patients with functional neurological symptom disorder (FND): Clinical pearls for front line providers

**DOI:** 10.1017/S1092852926101229

**Published:** 2026-10-23

**Authors:** Alison Buchholz, Jason Chua, Mackenzie Cervenka, Eva Keatley

**Affiliations:** 1 Psychiatry & Behavioral Sciences, Johns Hopkins School of Medicine, USA; 2 Neurology, Johns Hopkins School of Medicine, USA; 3 Physical Medicine and Rehabilitation, Johns Hopkins School of Medicine, USA

**Keywords:** Conversion disorder, dissociative seizures, functional movement disorder, functional neurological disorder, functional neurological, symptom disorder, functional seizures, nonepileptic seizures, psychogenic nonepileptic seizures

## Abstract

Our understanding of functional neurological symptom disorder (FND) has evolved more quickly than dissemination of our progressed knowledge. Healthcare providers remain suboptimally educated about and trained in FND-informed care. As a result, patients continue to have harmful interactions with the healthcare system around FND, often beginning with poorly established and delivered diagnosis and uninformed or otherwise lacking treatment planning. This results in iatrogenic harm to patients and misuse of healthcare resources. Appreciating this, providers repeatedly request guidance on FND-informed diagnosis and treatment. This article serves to provide guidance on FND diagnosis and treatment planning for healthcare providers and patients.

## Introduction

Functional Neurological Symptom Disorder (FND) is among the most common presentations to neurology clinics.^1^ Patients with FND overwhelmingly report negative experiences with healthcare professionals,^2^ and frontline providers are often the first to communicate the FND diagnosis to patients.^3^ There is burgeoning scholarly interest in FND over the last decade, and emphasis on positive signs for diagnosis improves confidence in diagnostic accuracy^4^. However, few providers receive training on making and communicating this complex diagnosis with psychiatric and neurological origins, which is often surrounded by uncertainty and stigma.^2^ As such, there is a critical unmet need for improved education and training among providers to reduce errors and destigmatize FND.^5^ Since conveying a clear diagnosis and explanation of FND is an essential first step in recovery,^2^ and until the Functional Neurological Disorder Society (FNDS) produces related consensus recommendations, this publication aims to provide recommendations on effective delivery of the FND diagnosis, as well as information for providers and patients regarding FND and its treatment options. Importantly, research is needed to better understand how incorporating these recommendations can improve outcomes, whether/how these recommendations should be modified to fit individual patient needs, and if/how such modifications can improve outcomes.

## Brief review of FND diagnostic criteria, per DSM-5-TR

FND, historically known as conversion disorder, includes multiple symptom presentations, including seizure-like episodes and involuntary movements. Per the Diagnostic and Statistical Manual of Mental Disorders, Fifth Edition, Text Revision (DSM-5-TR)^6^, FND is diagnosed based on the following criteria:

- One or more symptoms of altered voluntary motor or sensory function.
- Clinical findings provide evidence of incompatibility between the symptom and recognized neurological or medical conditions.
- The symptom or deficit is not better explained by another medical or mental disorder.
- The symptom or deficit causes clinically significant distress or impairment in social, occupational, or other important areas of functioning or warrants medical evaluation.

Clinical subtypes can be specified by:

- Symptom: weakness/paralysis, abnormal movement, swallowing, speech, attacks/seizures, anesthesia/sensory loss, special sensory (e.g., visual, olfactory, or hearing disturbance), or mixed
- Duration: acute <6 months, persistent >6 months
- Psychological stressor: with or without

## Differential considerations

Per the DSM-5-TR, FNDs are distinct from other somatic disorders, including somatic symptom disorder (preoccupation with and disability surrounding symptoms), illness anxiety disorder (preoccupation with having or acquiring an illness, in the absence of symptoms), factitious disorder (falsification of symptoms absent external incentive/gain), and malingering (falsification of symptoms with/for external incentive/gain) (DSM-5-TR, 2022)^6^. Distinguishing FND, especially from factitious disorder or malingering, can be challenging. The absence of legal proceedings or obvious incentives/gains, and if symptoms persist across settings and do not only occur when being examined by a medical provider, argue against factitious/malingering. In general, malingering in medical/non-legal contexts is quite low, with studies suggesting around 8%.^7^

## Introduction to tip sheets

Patients with FND can benefit from clear communication of diagnosis and treatment plans and from therapies. Ultimately, patients diagnosed with FNDs are often referred to reprogramming therapies, including psychotherapy and physical therapy. Neuropsychologists assist with differential diagnosis and treatment planning, but are also left to clarify and explain the FND diagnosis, given the lack of training for most medical professionals.

In our experience, referring providers often request assistance with delivery and explanation of the FND diagnosis and treatment options. To address this, we have developed the following four tip sheets, based on specific questions and issues often asked by patients and referring providers. The first two tip sheets are designed for FND with attacks or seizures (also known as functional seizures (FS)), with the first being for patients and the second being for medical providers. The last two tip sheets are intended for FND with abnormal movement (also known as functional movement disorders (FMD)), with the first being for patients and the second being for medical providers.

### 1. Tip sheet for patients diagnosed with functional neurological symptom disorder with attacks or seizures (also known as functional seizures (FS))

- What are functional seizures (FS)**?**
  - FS, historically referred to as pseudoseizures, conversion disorder, dissociative seizures, or psychogenic-non epileptic seizures (PNES), are a subtype of FND characterized by events with symptoms like epileptic seizures (for example, body shaking, inability to move or respond to others, feeling “out of body”), but are not due to abnormal electrical activity in the brain detected by scalp EEG.
  - FS are common and affect about 1/3 of people in an epilepsy monitoring unit (EMU).
  - FND, including FS, is a “real” condition and does not mean that you are “crazy” or that you are “faking” symptoms.
- How are FS diagnosed?
  - FS are diagnosed by continuous video electroencephalography (cvEEG) during episodes/attacks, ideally captured in an epilepsy monitoring unit (EMU), EEG laboratory, or at home using continuous video EEG monitoring. When these technologies are not available, a video or description of the episodes/attacks is used for diagnosis.
  - A normal EEG during episodes and between episodes confirms that episodes are most likely not epileptic.
  - If your neurologist has concerns that there could be another medical condition contributing to your episodes, for example, heart problems, they will refer you to the appropriate provider. If not, they will diagnose you with FS.
  - Some patients have both functional neurological symptom disorder (FND) and other neurologic problems, like epileptic seizures. Your neurologist can help determine if you have other neurologic problems in addition to FND.
- Why Do I Have FS?
  - We do not yet fully understand what causes FS at an individual level, but studies show evidence of differences in the connections between the parts of the brain involved in self-agency (i.e., sense of self-control), emotion regulation, sensory processing, and motor function in people with FND. For some patients, stressors seem to lead to the development and maintenance of symptoms, but that is not always the case.
  - Psychologists and neuropsychologists trained in FS can help inform why a given person might have FS and what type of treatment might be most helpful.
- Will I get better?
  - Most patients with FS get substantially better if they participate in the type of therapy that is recommended for them.
- What do I do now that I have been diagnosed?
  - It is sometimes helpful for patients to go to websites such as those listed below to read, hear, and see more about FND. Doing so can help with understanding and accepting the diagnosis, which is a critical step to getting better. We recommend the following:
    - https://fndhope.org/ and http://www.neurosymptoms.org/ are excellent resources for information on all types of FND and include specific sections on FS.
    - http://www.nonepilepticattacks.info/index.html and https://www.epilepsy.com/diagnosis/imitators-epilepsy/psychogenic-nonepileptic-seizures are good resources for information specifically about FS.
    - To find providers who specialize in FND, you can go to the following websites: https://fndhope.org/; https://www.fndsociety.org/referral/; https://nonepilepticseizures.com/
- What do I do while I am waiting to be seen by a specialist?
  - Psychotherapy can help reprogram/retrain the brain to reduce FND symptoms and can lead to improvements in daily functioning, quality of life, and associated problems, like anxiety and depression. While cognitive-behavioral therapy (CBT) can be helpful for FND, there are other forms of psychotherapy, including psychodynamic, dialectical behavior therapy (DBT), and hypnosis, which have shown promise for use in FND. Psychotherapy providers can be found by going to https://www.psychologytoday.com/us or http://locator.apa.org/. You can also call your medical insurance company to request a listing of mental health providers in your area.
  - Work with your psychotherapist to track triggers for your symptoms. If/when you think you found a trigger, work with your psychotherapist to determine if/how you can control, modify, avoid, or otherwise cope with the trigger.
  - Consider and discuss practicing distraction, grounding exercises, relaxation breathing and/or progressive muscle relaxation with your psychotherapist.
  - Consider the “Taking Control of Your Seizures” workbook^8^ and therapist guide,^9^ part of a “Treatments That Work” series. Importantly, the workbook should only be used with the guidance of a psychotherapist who is comfortable providing the treatment, per the “Therapist Guide.”
  - Consider the “Overcoming Functional Neurological Symptoms: a five areas approach” book.^10^

### 2. Tip sheet for providers diagnosing functional neurological symptom disorder with attacks or seizures (also known as functional seizures (FS))

- Know that Continuous video electroencephalography (cvEEG) in an ambulatory setting or an epilepsy monitoring unit (EMU) is considered the gold-standard diagnostic procedure for diagnosing FND with attacks or seizures. When cvEEG is not possible, a diagnosis of probable functional seizures may be based on history, semiology, and interictal EEG. Likewise, a diagnosis of clinically established functional seizures can be based on history, semiology, ambulatory EEG capture of a typical event (without video), and separate video recording of a typical event.^11^
- Know that a patient having functional seizures does not preclude the possibility of other medical, including neurologic, problems, such as epilepsy. As such, obtaining cvEEG of all typical seizure-like episodes where feasible is recommended.^11^
- If it is suspected that patients have another medical condition contributing to their suspected functional symptoms, that should be evaluated by an appropriate provider prior to diagnosing FND. If a patient has both FND with attacks or seizures and epilepsy, please explain that this occurs in 5%–20% (depending on source) of patients and that EMU admission is a critical step in differentiating events and informing treatment.
- Appreciate that your delivery of the diagnosis is a critical step in the multi-step treatment process. This step is so important that sometimes the delivery of diagnosis itself can result in improvements or even temporary elimination of events. At the same time, delivery that leaves patients feeling confused, stigmatized, or otherwise demoralized can result in rejection of the diagnosis and treatment attempts. When this happens, patients are more likely to continue to “doctor shop” in an attempt to find an underlying “medical cause” and unnecessarily overutilize medical resources.^11^
- Become comfortable with and use up-to-date terminology. Please consider using the term functional neurological symptom disorder (with attacks or seizures) or functional seizures instead of psychogenic non-epileptic seizures (PNES), non-epileptic seizures, pseudoseizures, and/or conversion disorder, and so forth. It might also be helpful to explain to patients (and their caregivers) that the term “seizure” is descriptive of behavior and does not necessarily imply abnormal electrical activity in the brain, as would be the case for someone with epileptic seizures.
- Explain how the FND diagnosis is made; clarify that it is not simply a diagnosis of exclusion (i.e., no abnormal activity on EEG during events) but that the onset, course, and semiology of the events are diagnostic.
- Attempt to explain the mechanism of the above as a “software” or “programming” problem as opposed to a “hardware” problem. Use this explanation as an opportunity to emphasize how psychotherapy can help reprogram the brain to experience, process, and express in a healthier way, which might result in fewer events (studies suggest on average 55%–60% reduction) and subsequent improvements in quality of life.
- Emphasize that some but not all people who develop FND have histories of trauma and psychiatric problems, and that some but not all experience a stressor prior to the onset of their events.
- Clarify that FND does not imply that the patient is faking their symptoms for some gain (e.g., to get out of work, to get disability benefits). Reiterate that the diagnosis of FND implies that they are not “faking.”
- Be honest about the evolving nature of our understanding of these disorders. Reiterate that despite our incomplete understanding of exactly how these symptoms manifest, the misdiagnosis rate of FND is very low (studies suggest 4%) and that this is likely even lower when the diagnosis is made following gold-standard procedures (e.g., cvEEG).
- Emphasize that FND is a very common diagnosis in neurology clinics.
- Inform the patient that referral to psychology might be helpful in elucidating why symptoms developed and what the focus of treatment should be. While CBT has the most empirical evidence as a potential theoretical and practical framework for psychotherapy for FND, there are numerous other forms of psychotherapy, including psychodynamic approaches, DBT, hypnosis, and neurobehavioral therapy (NBT) that have shown promise and are being investigated for use in FND.^12^
- Practice delivery and model this for learners if you are providing care in an academic setting, or select medical providers most adept at delivering this information to provide training to learners.

### 3. Tip sheet for patients diagnosed with functional neurological symptom disorder with abnormal movement, (also known as functional movement disorder (FMD))

- What is functional movement disorder (FMD)?
  - FMD is a disorder of the voluntary motor or sensory system with genuine symptoms that may include weakness, paralysis, tremor, dystonia, sensory disturbance, or any other symptoms impacting movement.
  - Symptoms may be constant or come and go.
- How is FMD diagnosed?
  - Ideally, diagnosis is made by a neurologist following a thorough workup. Neurologists look for positive signs that demonstrate impaired voluntary movement or sensation in the presence of intact automatic movement or sensation. Examples include symptoms that can change or resolve when attention is diverted to something else, bypassing the voluntary movement networks. It is important that your providers help you recognize and identify these positive signs throughout the diagnostic process so you can understand the condition. You are encouraged to ask any provider who is diagnosing FMD what they observed that led to the diagnosis.
- Why do I have FMD?
  - FMD develops due to a combination of psychological and neurological factors. FMD often occurs because the brain is sending faulty signals to the body. In other words, there is a problem with the way your brain is communicating with your muscles, leading to abnormal movements.
  - The good news is that extensive medical workup has shown there is no neurological damage that is causing your symptoms. The hardware (your nerves and muscles) is ok, but the software (signaling) is faulty. Since we are unable to capture this faulty signaling with current clinically used imaging techniques, it can be hard to understand and explain. There is also much we do not yet understand about why some people develop FMD, and others do not.
  - It may take time to understand how the diagnosis applies to you, and getting input from providers with various forms of expertise, including psychologists, can be helpful.
- Why do providers keep telling me I’m stressed?
  - The relationship between stress and FMD is complicated and sometimes the message gets oversimplified. We know that FMD symptoms are often triggered by a physically (e.g., injury or illness) or emotionally stressful event. But sometimes symptoms seem to come out of nowhere without any clear trigger. While stress may play a role, it hardly explains the whole picture. While some people notice a clear relationship between psychological stress (“I’m feeling overwhelmed!”) and their symptoms, others report no relationship at all. Working with rehabilitation therapists and psychologists can help with better understanding how the relationship between stress and symptoms applies to you.
- What do I do now that I have been diagnosed?
  - It is sometimes helpful for patients to go to websites such as those listed below to read, hear, and see more about FMD. Doing so can help clarify the diagnosis and increase acceptance of the diagnosis, which is a critical step to getting better. We recommend the following:
    - https://fndhope.org/ and http://www.neurosymptoms.org/ are excellent resources for information on all types of FND and include specific sections on FMD.
    - To find providers who specialize in FND, you can go to the following websites: https://fndhope.org/; https://www.fndsociety.org/referral/
- Will working with a mental health provider help?
  - Mental health providers such as psychologists, neuropsychologists, psychiatrists, neuropsychiatrists, and psychotherapists can be very helpful in learning about the FMD diagnosis, how it applies to you, and how to treat and manage your symptoms while continuing to pursue a high-quality of life. Treatment with a psychologist should be tailored to your needs and may include CBT among other therapeutic techniques. Although mental health providers who specialize in FND can be hard to find, there are many things you can do while waiting for specialty care as outlined below.
- Will rehabilitation therapies help?
  - Evidence points to rehabilitation therapies (i.e., physical therapy, occupational therapy) being among the most effective treatments available. It is important to learn and understand core concepts of FMD during treatment such as the role of attention in making symptoms better or worse, relaxation strategies to facilitate ease with movement, and identifying factors that facilitate movement (e.g., good sleep) or hinder movement (e.g., looking at feet, overthinking a movement, fatigue).
- What do I do while I am waiting to be seen by a specialist?
  - When specialists are not available, meeting with a psychotherapist to help with coping, adjustment to symptoms, and stress management can go a long way to improve quality of life. Consider these resources: www.psychologytoday.com and http://locator.apa.org/
  - Below are recommended books to learn about and manage FND symptoms:
    - “Overcoming functional neurological symptoms: a five areas approach”.^10^
    - “Reset & Rewire: The FND Workbook for Kids & Teens”.^13^ Designed for teenagers, this book has many elements that apply to people of all ages learning about FND.

### 4. Tip sheet for providers diagnosing functional neurological symptom disorder with abnormal movement (also known as functional movement disorder (FMD))

- Use up-to-date terminology when discussing the diagnosis with patients, such as functional movement disorder and functional neurological disorder with abnormal movements. Avoid terms like psychogenic, conversion, or hysteria. Explain that FMD is a subtype of FND.
- FMD should be diagnosed by a physician following a physical exam, history, and workup and be based on positive signs of FMD.
- FMD is not a diagnosis of exclusion. Positive signs can be assessed at bedside or in clinic and must be present for diagnosis. Examples of positive signs include Hoover’s sign, hip abductor sign, or symptoms that are distractible or resolve when attention is diverted. Know that these signs are sensitive and specific. More information on how to do an assessment can be found in the literature (e.g.,^4^).
- Inform patients that FMD is a common diagnosis in neurology and that population prevalence rates are estimated to be 4–5/100000.
- Be explicit that FMD symptoms are real, and the diagnosis does not imply that the patient is faking their symptoms for some gain (e.g., to get out of work, to get disability benefits). Reiterate that the diagnosis of FND implies that they are not “faking.”
- Know that a patient with FMD may also have other medical and neurologic problems. If patients have another medical condition contributing to their symptoms, it should be evaluated by an appropriate provider. Sometimes FMD is diagnosed along with a comorbid neurologic condition.
- When diagnosing FMD, demonstrate to patients how the diagnosis is being made, highlight how their symptoms are distractible, inconsistent, or unlike symptoms from other disorders being considered. This will help with diagnostic buy-in from the patient, which is important for recovery.
- Explain to patients that FMD is due to the brain sending faulty signals to the body. Below are some common metaphors to help explain FMD:
  - **Hardware/Software metaphor:** *“The good news is that extensive medical work up has shown there is no neurological damage that is causing your symptoms. Instead, we think there is a problem with the way your brain is sending signals to your muscles. The hardware (your nerves and muscles) is ok. But the software, the motor program your brain is sending, is glitchy. Of course, we can’t see that on structural imaging. So, there’s nothing wrong with the computer or the wiring, but some buggy software is causing problems in the way your body moves. We need to reprogram the system to help it work again.”*
  - **Broken picker metaphor:** *“Although movement usually feels automatic, it’s actually a two-step process. First your brain creates plans for the movement. Some are good, and some are not. Then it picks the best plan and sends that information to your muscles. This happens almost instantly. In FMD, the brain’s plan selector is picking the wrong plan, often resulting in abnormal movements.”*
- Explain that some people with FMD have experienced psychological or medical stressors prior to symptom onset, but this is not true for all patients with FMD. Explain that the relationship between FMD and stress is complicated and different for everyone. Avoid overemphasizing the role of stress, as this often does not resonate with patients and harms credibility and diagnostic acceptance.
- While FMD can be a chronic condition, it is not permanent, and symptoms can wax and wane over time and sometimes go away forever. Instill hope that treatment can work, and recovery is possible.
- Evidence points to psychological intervention and rehabilitation therapies (e.g., physical therapy) being the most effective treatments available, although outcomes will likely vary across individuals. These approaches can help patients better understand their FMD, improve independent functioning, and resolve abnormal movements.
- Partnering with psychologists and psychiatrists can be very helpful with differential diagnosis and treatment planning. When possible, collaborate with colleagues in psychology and psychiatry to improve patient outcomes.
